# Mutant TDP-43 does not impair mitochondrial bioenergetics in vitro and in vivo

**DOI:** 10.1186/s13024-017-0180-1

**Published:** 2017-05-08

**Authors:** Hibiki Kawamata, Pablo Peixoto, Csaba Konrad, Gloria Palomo, Kirsten Bredvik, Meri Gerges, Federica Valsecchi, Leonard Petrucelli, John M. Ravits, Anatoly Starkov, Giovanni Manfredi

**Affiliations:** 1 Feil Family Brain and Mind Research Institute, Weill Cornell Medicine, 407 East 61st Street, RR507, New York, NY 10065 USA; 2 Department of Natural Sciences, CUNY Baruch College, New York, NY USA; 3 Department of Neuroscience, Mayo Clinic, Jacksonville, FL USA; 4 Department of Neuroscience, University of California San Diego, La Jolla, CA USA

**Keywords:** TDP-43, TAR DNA-binding protein 43, Mitochondria, Bioenergetics, Calcium, ALS

## Abstract

**Background:**

Mitochondrial dysfunction has been linked to the pathogenesis of amyotrophic lateral sclerosis (ALS) and frontotemporal lobar degeneration (FTLD). Functional studies of mitochondrial bioenergetics have focused mostly on superoxide dismutase 1 (SOD1) mutants, and showed that mutant human SOD1 impairs mitochondrial oxidative phosphorylation, calcium homeostasis, and dynamics. However, recent reports have indicated that alterations in transactivation response element DNA-binding protein 43 (TDP-43) can also lead to defects of mitochondrial morphology and dynamics. Furthermore, it was proposed that TDP-43 mutations cause oxidative phosphorylation impairment associated with respiratory chain defects and that these effects were caused by mitochondrial localization of the mutant protein. Here, we investigated the presence of bioenergetic defects in the brain of transgenic mice expressing human mutant TDP-43 (TDP-43^A315T^ mice), patient derived fibroblasts, and human cells expressing mutant forms of TDP-43.

**Methods:**

In the brain of TDP-43^A315T^ mice, TDP-43 mutant fibroblasts, and cells expressing mutant TDP-43, we tested several bioenergetics parameters, including mitochondrial respiration, ATP synthesis, and calcium handling. Differences between mutant and control samples were evaluated by student t-test or by ANOVA, followed by Bonferroni correction, when more than two groups were compared. Mitochondrial localization of TDP-43 was investigated by immunocytochemistry in fibroblasts and by subcellular fractionation and western blot of mitochondrial fractions in mouse brain.

**Results:**

We did not observe defects in any of the mitochondrial bioenergetic functions that were tested in TDP-43 mutants. We detected a small amount of TDP-43^A315T^ peripherally associated with brain mitochondria. However, there was no correlation between TDP-43 associated with mitochondria and respiratory chain dysfunction. In addition, we observed increased calcium uptake in mitochondria from TDP-43^A315T^ mouse brain and cells expressing A315T mutant TDP-43.

**Conclusions:**

While alterations of mitochondrial morphology and dynamics in TDP-43 mutant neurons are well established, the present study did not demonstrate oxidative phosphorylation defects in TDP-43 mutants, in vitro and in vivo. On the other hand, the increase in mitochondrial calcium uptake in A315T TDP-43 mutants was an intriguing finding, which needs to be investigated further to understand its mechanisms and potential pathogenic implications.

## Background

Mutations in transactivation response element DNA-binding protein 43 (TDP-43) cause rare forms of familial amyotrophic lateral sclerosis (fALS) and frontotemporal lobe dementia (FTLD) [1, 2]. The incidence of TDP-43 mutations is estimated at approximately only 4% of all familial cases of ALS. However, abnormal TDP-43 cytosolic aggregates in motor neurons are a common pathological feature of the majority of sporadic and familial ALS autopsies [3], suggesting that even in the absence of mutations, TDP-43 may be involved in disease pathogenesis.

TDP-43 is a nuclear riboprotein involved in regulation of RNA splicing [4]. Under physiological conditions, TDP-43 localization is predominantly nuclear, but under stress TDP-43 translocates to the cytoplasm, where it localizes to stress granules. Mutant TDP-43, or even wild type protein when it is overexpressed, can form ubiquitin-positive aggregates in the extra-nuclear cell compartment.

In ALS-FTLD, mutant TDP-43 is depleted from the nucleus and accumulates in the cytosol, where it forms aggregates of phosphorylated protein [3]. Therefore, the pathogenic mechanisms of mutant TDP-43 may include both loss of nuclear function and gain of extra-nuclear toxic functions. Indeed, numerous lines of evidence support a role for extra-nuclear TDP-43 in ALS-FTLD [5, 6], and there is a strong possibility that TDP-43 oligomers spread from cell to cell in a prion-like fashion [7].

Mitochondria have been suggested to be one of the multiple potential targets of TDP-43 extra-nuclear mislocalization and aggregation, since mitochondrial dynamics and distribution has been described in cellular and animal models of the disease. In particular, it was shown that both TDP-43 overexpression and suppression impair mitochondrial movement in cultured neurons and that mutant TDP-43 co-localizes with mitochondria [8]. Furthermore, mitochondrial accumulation and aggregation was found in various TDP-43 animal models, including the transgenic mice expressing C-terminal fragments [9] or full-length protein [10]. In these models, mitochondrial aggregates in the cell bodies of motor neurons were accompanied by depletion of mitochondria in motor terminals and neuromuscular junctions, suggesting that mitochondrial axonal transport was impaired.

Mitochondrial axonal transport defects were identified in various TDP-43 animal models, from flies [11] to mice [12], and are likely to contribute to mitochondrial mislocalization and possibly to neuronal functional defects and degeneration. Interestingly, cytosolic localization of TDP-43 has also been linked to alterations in the interactions between mitochondria and endoplasmic reticulum [13], leading to functional defects, predominantly in intracellular calcium handling, which could also have important implications for ALS-FTLD pathogenesis.

Recently, it was proposed that mutant cytosolic TDP-43 gains access to the mitochondrial matrix through a dysregulated import mechanism, and that TDP-43 in the matrix impairs mitochondrial respiratory chain activity by downregulation of complex I biosynthesis [14].

Based on the growing body of evidence suggesting that mitochondria are targeted by mutant TDP-43, we decided to perform a thorough investigation of mitochondrial bioenergetics in multiple model systems, ranging from mutant TDP-43 transgenic mice to patient-derived cells harboring TDP-43 mutations, to asses the presence of mitochondrial dysfunction.

## Methods

### Chemical reagents

All reagents were from Sigma-Aldrich (St. Louis, MO) unless otherwise stated.

### Animals

We used the strain B6;CB-Tg(Prnp-TARDBP*A315T)95Balo/J of TDP43^A315T^ transgenic mice (from The Jackson Laboratory, Bar Harbor, ME). In all experiments, N-Tg littermates were used as controls.

The criterion for determining disease onset was the development of abnormal hind-limb extension (clasping). The criterion for survival was the inability of the mouse to right itself, when placed on its side (loss of righting reflex).

### Bioenergetics and calcium uptake measurements in brain mitochondria

Forebrain mitochondrial fractions were freshly prepared from TDP43^A315T^ transgenic mice and age and sex matched N-Tg littermates by differential centrifugation of homogenates on a discontinuous Percoll™ gradient as previously described [15, 16]. Mitochondria were obtained from the non-synaptosomal gradient layer and washed 3 × in buffer containing 75 mM sucrose, 225 mM mannitol, 10 mM HEPES; 2 mM EDTA pH 7.4.

ATP synthesis was measured in purified brain mitochondria using a luciferin-luciferase approach, as previously described [17]. Glutamate (5 mM) and malate (2 mM) or succinate (5 mM) plus rotenone (1 μM) were used as oxidative substrates. Measurements were carried out by luminometry.

ROS emission was measured as Amplex Red (Thermo Fisher Scientific, Waltham, MA) fluorescence (555 nm excitation and 581 nm emission wavelengths) in the presence of exogenous horseradish peroxidase and mitochondrial H_2_O_2_ as described [18, 19]. Briefly, 100 μg mitochondria were added to 1 mL incubation buffer (125 mM KCl, 20 mM HEPES, 0.2 mM EGTA, 2 mM KH_2_PO_4_, 200 μg/mL BSA, 1 μM Amplex Red, 4 U horseradish peroxidase, pH 7.2). Standard curves were used to calculate H_2_O_2_ emission rates after sequential addition of substrate (5 mM glutamate, 2 mM malate), 1 μM rotenone, and 1.8 μM antimycin A.

Mitochondrial Ca^2+^ uptake was estimated fluorimetrically with Fura 6 (340/380 nm excitation and 510 nm emission wavelengths) (Thermo Fisher Scientific) with sequential additions of 25 nmoles of Ca^2+^ to the incubation medium (125 mM KCl, 20 mM Hepes, 1 mM MgCl_2_, 2 mM KH_2_PO_4_, 0.2 mM ATP, 1 μM rotenone, 5 mM succinate, 0.3 μM Fura 6, pH 7.2).

Mitochondrial membrane potential was estimated using safranin O (excitation and emission wavelengths of 495 nm and 586 nm, respectively), as previously described [15]. The incubation buffer contained 125 mM KCl, 20 mM HEPES, 1 mM MgCl_2_, 2 mM KH_2_PO_4_, 0.2 mM ATP, 200 μg/mL BSA, 5 mM glutamate, 2 mM malate, 2 μM safranin O, pH 7.2). Mitochondrial membrane potential decay curves were obtained by repetitive additions of 25 nmol Ca^2+^ or 2–16 nM of the respiratory chain uncoupler SF6847.

For mitochondrial respiration, 100 μg of brain mitochondria were resuspended in 0.5 ml of respiration buffer containing 125 mM KCl, 20 mM Hepes, 4 mM K_2_HPO_4_, 0.1 mg/ml BSA, pH 7.2 and 1 mM ADP. Glutamate (5 mM) and malate (2 mM) were used as oxidative substrates. Oxygen consumption was recorded with an oxygraph equipped with a Clark electrode (Hansatech, Norfolk, UK), as described [20], before (state 4) and after the addition of ADP (state 3). SF6847 (0.1 μM) was used to fully uncouple mitochondrial respiration (state 3 uncoupled).

Respiratory chain complex I and complex IV enzymatic activities were measured spectrophotometrically, as previously described [21].

### Bioenergetics measurements in cultured cells

Skin fibroblasts (from the repository at the University of California, San Diego) were cultured in Dulbecco modified Eagle medium (DMEM) supplemented with 25 mM glucose, 4 mM glutamine, 1 mM pyruvate, and 10% fetal bovine serum. All fibroblast lines were coded to protect patients’ identity.

HEK293T (from American Type Culture Collection, ATCC, Manassas, VA), were grown in DMEM supplemented with 25 mM glucose, 4 mM glutamine, 1 mM pyruvate, and 5% fetal bovine serum.

All cells tested negative for mycoplasma contamination by PCR assays of the culture medium using previously described primer sets and amplification protocols [22].

For mitochondrial membrane potential and mitochondrial content measurements skin fibroblasts were seeded at the density of 1.5 × 10^4^ cells/well in replicates of eights in 96-well tissue culture plates in growth medium incubated at 37 °C in 5% CO_2_. The following day, all cells were washed with cultured medium and loaded with 50 nM tetramethylrhodamine methyl ester (TMRM; 544ex, 590em; Thermo Fisher Scientific) and 450 nM Mito Tracker Green (MTG; 490ex, 516em; Thermo Fisher Scientific) for 30 min at 37 °C in phenol-free DMEM containing 5 mM glucose, 4 mM glutamine, and 1 mM pyruvate, half of the wells additionally contained the protonophore carbonyl cyanide p-trifluoromethoxyphenylhydrazone (FCCP; 2 μM) to completely depolarize mitochondria and obtain background TMRM and MTG fluorescence, which were subtracted from total fluorescence levels. After washing with DMEM, MTG and TMRM fluorescence were simultaneously recorded in a plate reader equipped with a polychromator (Spectramax 5; Hitachi, Tokyo, Japan). MTG and TMRM fluorescence values were expressed as relative fluorescence units per milligram of total cellular proteins (DC Protein Assay; Bio-Rad, Hercules, CA).

Total ATP content in fibroblasts was measured by luciferase reactions in a luminometry plate reader, according to the manufacturer’s guidelines (Promega, Madison, WI). Cells were seeded at the density of 1.5 × 10^4^ cells/well in replicates of nines in 96-well tissue culture plates in growth medium incubated at 37 °C in 5% CO_2_. On the following day, triplicates of wells were incubated with either DMEM containing 5 mM glucose, 4 mM glutamine, and 1 mM pyruvate (ATP baseline), DMEM containing 5 mM 2-Deoxy-D-glucose (2DG), 4 mM glutamine, and 1 mM pyruvate to block glycolysis (ATP 2DG) or DMEM containing 5 mM glucose, 4 mM glutamine, 1 mM pyruvate, and 1 μM oligomycin to block the mitochondrial ATPase (ATP Oligo) for 90 min. Cells were washed with PBS and lysed in 30 μl of trichloroacetic acid (2.5% *W*/*V*) on ice for 30 min. Following lysis, 20 μl aliquots were pipetted into a separate plate for protein determination for data normalization, and 45 μl of Tris-acetate (400 mM, pH 8.0) buffer was added to each well of the remaining lysates. Bioluminescence was measured promptly after adding and mixing 20 μl of luciferase reagent. Luminescence values were normalized against an ATP standard and normalized by protein content.

Oxygen consumption rate (OCR) and extracellular acidification rate (ECAR) were measured in human fibroblasts or TDP-43 transfected HEK293T cells with a Seahorse XF96 Flux Analyzer (Agilent, Santa Clara, CA). Cell lines were seeded in 12 wells of a XF 96-well cell culture microplate at a density of 1 × 10^4^ cells/well (fibroblasts) and 2 × 10^4^ cells/well (HEK293T cells) in 200 μL of growth medium and incubated for 24 h at 37 °C in 5% CO_2_. After replacing the growth medium with 200 μL of XF Assay Medium supplemented with 5 mM glucose, 1 mM pyruvate and 4 mM glutamine, pre-warmed at 37 °C, cells were degassed for 1 h before starting the assay procedure, in a non-CO_2_ incubator. OCR and ECAR were recorded at baseline followed by sequential additions of 1 μM oligomycin, 2 μM FCCP, and 0.5 μM Antimycin A (AA) plus 0.5 μM Rotenone (Rot). Non-mitochondrial oxygen consumption (in the presence of AA + Rot) was subtracted from all OCR values, and outliers technical replicates outside the 2 standard deviation of the mean were discarded for both ECAR and OCR. In fibroblasts, values were normalized by the mean protein value of each line measured by modified Lowry protein assay. In HEK293T cells, values were normalized by total DNA content measured by fluorescence of DAPI stain, using DNA standards for quantification.

### Cultured cells transfection and immunostaining

HEK293T or HeLa cells were transiently transfected with plasmids expressing recombinant WT or mutant human TDP-43-myc or empty vector, using FuGENE-HD (Promega), according to the manufacturer’s protocol. For ER and mitochondrial contact analyses, HeLa cells were co-transfected with ddGFP plasmids targeted to mitochondria and ER (Tom20-ddGFP and calN-ddGFP, respectively) together with DSred2-Mito. Cells were imaged 48 h later on the Leica TCS SP5 confocal microscope on a live imaging stage, controlled at 37 °C.

Live cell calcium imaging was performed with HeLa cells co-transfected with WT or mutant TDP-43 and mitochondrially targeted GCamp6. 48 h after transfection, cells were perfused with 20 μM ATP in imaging medium containing (156 mM NaCl, 3 mM KCl, 2 mM MgSO_4_, 1.25 mM KH_2_PO_4_, 10 mM glucose, 1 mM EGTA and 10 mM HEPES, pH 7.35), followed by replenishment of ER calcium with 2 mM calcium to induce store operated calcium entry, as described previously [23].

### Brain mitochondria isolation for TDP-43 western blots

Brains from TDP43^A315T^ transgenic mice and littermate N-Tg controls were cryopreserved by cutting the whole brain into 30–40 mg sections, placing them into solution containing 300 mM sucrose and 20% (by volume) DMSO, freezing in liquid nitrogen, and storing them at −80 °C until the experiment. For mitochondria isolation, ~100 mg of frozen brain tissue was slowly (1 h) thawed on ice, then the cryopreservation solution was replaced with homogenization medium (HM) comprising 225 mM sucrose, 75 mM mannitol, 1 mM EGTA, 20 mM HEPES (pH 7.4), and 1 mg/ml bovine serum albumin essentially fatty acid free (BSA). Brain tissue was homogenized in 10 ml of HM with a Dounce tissue grinder (15 ml volume, glass tube-glass pestle) manually by 40 strokes, in ice (step 1). All further procedures were performed at 4 °C. The homogenate was centrifuged at 2000 g × 5 min; the supernatant was further centrifuged at 12000 g ×10 min (step 2). The pellet from this step was collected, resuspended in 1 ml of HM, loaded on top of 9 ml of 23% Percoll™ separation medium, and centrifuged at 31,000 g × 10 min. The Percoll™ separation medium was prepared by dissolving 225 mM sucrose, 75 mM mannitol, 1 mM EGTA, 20 mM HEPES in 100% Percoll™ and adjusting pH to 7.4; this medium was diluted with HM to 23% Percoll™. The pellet from this step was collected and washed 2 times by centrifuging at 12000 g × 10 min in HM. The final pellet was dissolved in 200 μl of HM devoid of BSA (HM^-BSA^). For digitonin treatment, this pellet was thoroughly resuspended in 10 ml of HM^-BSA^ and 20 μl of 10% digitonin DMSO solution (0.02%, final digitonin concentration) was added to the suspension. After incubating on ice for 5 min, the suspension was centrifuged at 12000 g × 10 min, the pellet was resuspended in digitonin-free HM^-BSA^ and centrifuged again at 12000 g × 10 min. The final solid pellet was resuspended in 100 μl of HM^-BSA^ and stored frozen at −20 °C until the experiment.

For assaying combined synaptic + nonsynaptic mitochondria, the isolation procedure was modified as follows. The supernatant from step 2 above was collected, and treated with 0.02% digitonin for 5 min in ice, then centrifuged at 12000 g × 10 min. The pellet was resuspended in 10 ml of HM and centrifuged 12,000 g × 10 min. To remove proteins electrostatically attached to mitochondria membranes, the pellet from this step (contains synaptic + non-synaptic mitochondria) was resuspended in 2 ml of medium comprising 6 M KCl, 20 mM HEPES (pH 7.4) and incubated in ice for 5 min with occasional gentle agitation. The suspension was diluted with HM-BSA to 10 ml and centrifuged 12,000 g × 10 min, and the pellet was collected. To prepare digitonin -treated mitochondria from this fraction, mitochondria pellet was diluted to 10 ml with HM-BSA and treated with 0.01% digitonin for 7 min in ice, with occasional gentle agitation. The suspension was centrifuged 12,000 g × 10 min, the pellet was collected and washed in 10 ml of HM-BSA and centrifuging 12,000 g × 10 min; the procedure was repeated once. The final solid mitochondria pellet was resuspended in 100 μl of HM-BSA and stored frozen at −20 ^o^C until the experiment.

### Western blot analyses

Samples were lysed in RIPA buffer (Thermo Fisher Scientific) and centrifuged at 14,000 rpm at 4 °C for 20 min. Supernatants were diluted in 2X Laemmli sample buffer (Bio-Rad). Proteins were separated on 10 or 4–20% gradient Mini-PROTEAN TGX gels (Bio-Rad) and transferred to nitrocellulose blotting membranes (Bio-Rad). Membranes were probed with primary antibodies against FLAG tag (M2, Sigma), MCU (Sigma), MICU1 (Abcam, Cambridge, MA), cytochrome C (Cell Signaling, Danvers, MA), Cyclophilin D (Millipore, Billerica, MA), Tim23 (BD Biosciences, San Jose, CA), VDAC1 (Abcam), COX1 (Abcam), GAPDH (Thermo Fisher Scientific), citrate synthase (Abcam), and an oxidative phosphorylation (OXPHOS) cocktail (Abcam) overnight at 4 °C. Blots were then probed with horseradish peroxidase-conjugated anti-mouse (Jackson ImmunoResearch, West Grove, PA) or anti-rabbit (Thermo Fisher Scientific) secondary antibodies and detected using enhanced chemiluminescence (Bio-Rad).

### Statistical analyses

All data are presented as mean ± standard deviation in all experiments, except for the results of Figs. 4 and 5, which are presented as mean ± standard error of the mean. The results were compared using Student’s t-test or, when more than one condition was examined, one-way ANOVA with Bonferroni correction. In either case, a *p* value <0.05 was considered significant.

## Results

### Mitochondrial bioenergetics is unaffected in mutant TDP-43^A315T^ mouse brain

The B6.Cg-Tg(Prnp-TARDBP*A315T)95Balo/J mouse strain (from now on referred to as TDP-43^A315T^ mice) expresses human A315T mutant TDP-43 with a N-terminal FLAG tag in the nervous system, under the control of the prion promoter. The expression of the transgene results in a 3-fold increase in the levels of TDP-43 in the brain. These transgenic mice develop a neurodegenerative disease characterized by abnormal gait starting at 2 moths of age, TDP-43 and ubiquitin pathology in brain and spinal cord, and motor neuron degeneration [7]. In TDP-43^A315T^ mice, CNS degeneration is accompanied by degeneration of the myoenteric plexus, resulting in premature death due to intestinal paralysis [24]. The average survival of our cohort of TDP-43^A315T^ mice obtained from The Jackson Laboratories was 103.25 days (SD = 22.89 days, *n* = 20), consistent with previously reported survival data [7].

**Fig. 1:**
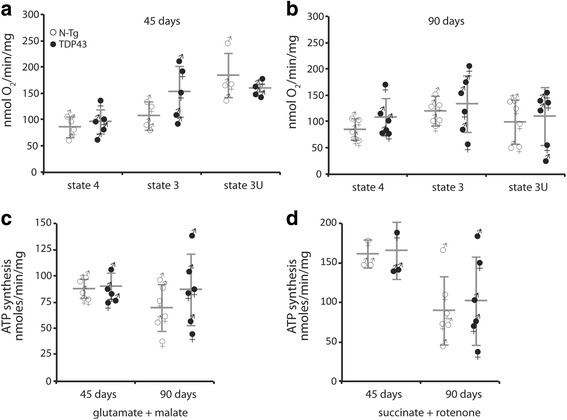
OXPHOS is unchanged in brain mitochondria of TDP-43 mutant mice. **a** Oxygen consumption measured in freshly isolated brain mitochondria of TDP-43^A315T^ mice (TDP-43) and non-transgenic (N-Tg) littermates at 45 days of age. Glutamate and malate were used as respiratory substrates. State 4 indicates non-stimulated respiration. State 3 (phosphorylating) respiration was induced by addition of ADP, followed by addition of FCCP to activate maximum uncoupled respiration (state 3 U). **b** Oxygen consumption measured as in (A) at 90 days of age. **c** Complex I dependent mitochondrial ATP synthesis with glutamate and malate in freshly isolated brain mitochondria of TDP-43^A315T^ mice and N-Tg littermates at 45 and 90 days of age. **d** Complex II dependent ATP synthesis with succinate as substrate, in the presence of the complex I inhibitor rotenone, at 45 and 90 days of age. In all panels, male and female samples in each group are indicated by the respective sex symbols. Average values are indicated by horizontal lines ± SD

Since TDP-43^A315T^ mice develop clear TDP-43 pathology in the brain [7], we tested whether brain mitochondrial bioenergetics was affected. Mitochondria were freshly isolated from adult mice brain at 45 days and at 90 days (presymptomatic and symptomatic ages, respectively). Oxygen consumption was measured using glutamate plus malate as substrates, which drive respiration through complex I. There were no respiratory defects in TDP-43A315T relative to non-transgenic (N-Tg) littermates brain mitochondria at 45 days (Fig. 1a) and at 90 days (Fig. 1b). No differences were found in state 4 respiration (non-phosphorylating) and state 3 uncoupled respiration (non-phosphorylating, after addition of the uncoupler FCCP).

Mitochondrial ATP synthesis was also measured in isolated mouse brain mitochondria at 45 and 90 days of age using a kinetic assay. ATP synthesis driven by complex I substrates malate plus glutamate did not differ between TDP-43^A315T^ and N-Tg littermates brain mitochondria at either ages (Fig. 1c). Similarly, complex II-driven ATP synthesis with succinate as substrate, in the presence of complex I inhibitor rotenone, did not differ in mutant and littermate control mice (Fig. 1d).

**Fig. 2:**
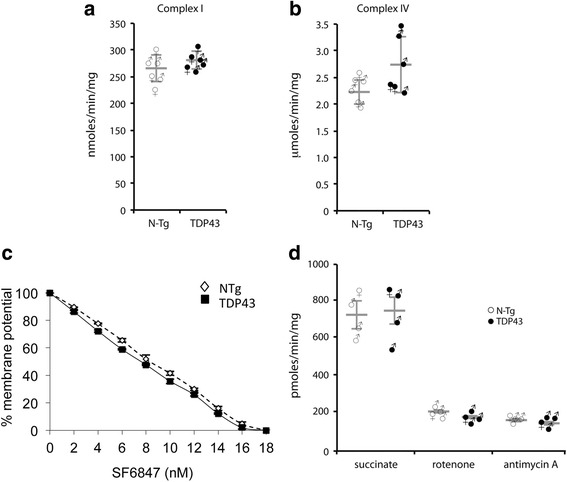
Respiratory chain activities, membrane potential, and ROS emission are unchanged in brain mitochondria of TDP-43 mutant mice. **a** Complex I activity (expressed as nmoles substrate/min/mg of mitochondrial protein) in brain mitochondrial membranes from TDP-43^A315T^ mice and N-Tg littermates at 60 days of age. **b** Complex IV activity in brain mitochondrial membranes from TDP-43^A315T^ mice and N-Tg littermates at 60 days of age. **c** Membrane potential measured with safranin O in intact freshly isolated brain mitochondria of TDP-43^A315T^ mice and N-Tg littermates at 60 days of age. Mitochondria were challenged with increasing concentrations of the membrane uncoupler SF6847. *n* = 6 mice in each group, 3 males and 3 females. **d** Hydrogen peroxide emission from intact freshly isolated brain mitochondria of TDP-43^A315T^ mice and N-Tg littermates at 60 days of age measured by Amplex red fluorescence

Since no defects in mitochondrial respiration and ATP synthesis were detected at presymptomatic (45 days) and at symptomatic (90 days) ages, in order to avoid potentially confounding effects due to major changes in brain cyto-architecture associated with neurodegeneration, we opted to continue the measurements of mitochondrial function at disease onset (60 days). The enzymatic activities (Vmax) of respiratory chain complexes I and IV (cytochrome oxidase) measured in isolated brain mitochondria (Fig. 2a, b) did not differ in TDP-43^A315T^ and N-Tg littermate brain mitochondria. Furthermore, we measured the sensitivity of brain mitochondrial membrane potential to increasing doses of the uncoupler SF6847, using safranin O in a fluorimetric assay. This assay is an indicator of the mitochondrial bioenergetic power, as the respiratory chain attempts to prevent loss of membrane potential by increasing electron transfer. The slopes of mitochondrial membrane depolarization in response to uncoupling (Fig. 2c) were virtually identical in mutant and littermate control mice at 60 days of age (*n* = 6 mice per group, 3 males and 3 females), further suggesting lack of bioenergetic differences.

Lastly, the rate of hydrogen peroxide emission from brain mitochondria respiring with succinate as substrate was measured by Amplex red fluorescence, under basal conditions and after sequential additions of rotenone and antimycin A to block respiratory chain complex I and complex II, respectively. H_2_O_2_ emission in all conditions did not differ in 60 days old TDP-43^A315T^ and N-Tg littermate brain mitochondria (Fig. 2d).

Taken together these results indicated that expression of mutant TDP-43 in mouse brain did not result in significant bioenergetic defects at ages that preceded or followed symptom onset in these mice.

### Mitochondrial calcium uptake is elevated in mutant TDP-43^A315T^ mouse brain

**Fig. 3:**
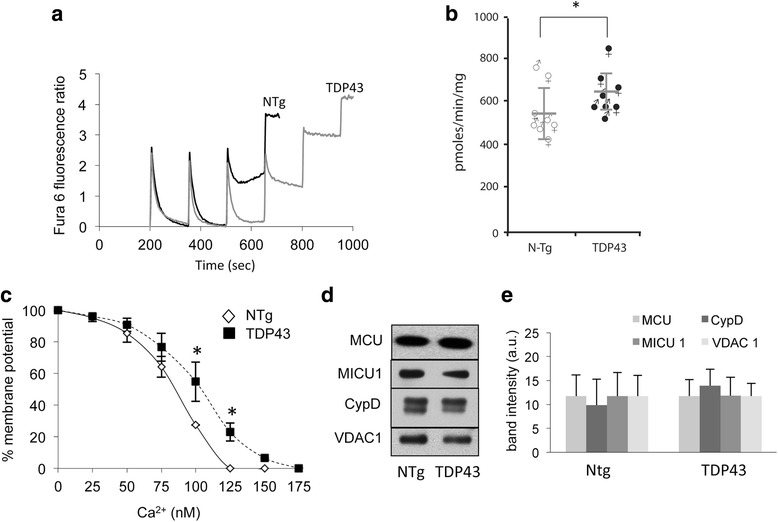
Brain mitochondrial calcium capacity is increased in TDP-43 mutant mice. **a** Representative brain mitochondrial calcium uptake curves. TDP-43^A315T^ mitochondria take up more calcium than N-Tg mitochondria, as shown by the higher number of bolus additions necessary to cause calcium uptake arrest (i.e., lack of deflection in the fluorescence curve). **b** Quantification of total brain mitochondrial calcium capacity in TDP-43^A315T^ and N-Tg mitochondria at 60 days of age. * *p* < 0.05. **c** Membrane potential measured with safranin O in intact freshly isolated brain mitochondria of TDP-43^A315T^ mice and N-Tg littermates at 60 days of age. Mitochondria were challenged with increasing concentrations of calcium. *n* = 6 mice in each group, 3 males and 3 females. * *p* < 0.05. **d** Representative western blots of mitochondrial proteins involved in calcium uptake (MCU, MICU1, CypD, VDAC1). **e** Quantification of band intensities from western blots. Values are arbitrary densitometric units. *n* = 9 and 8 mitochondrial preparations for N-Tg and TDP-43^A315T^, respectively. Equal amounts of mitochondrial proteins (2.5 μg) were loaded for each sample

The ability to buffer calcium is a fundamental property of brain mitochondria, which is altered in various mouse models of neurodegenerative diseases, including familial ALS associated with mutant SOD1 [15, 16, 25]. Therefore, we tested mitochondrial calcium capacity in isolated brain mitochondria of 60 days old transgenic and N-Tg mice using Fura 6 in a fluorimetric assay. After each bolus addition of calcium (20 nmoles), there was a rapid uptake as shown by a downward deflection of the fluorescence traces, reflecting calcium entry into mitochondria, from which Fura 6 is excluded. When the calcium capacity is saturated, mitochondria stop taking up calcium, indicated by a lack of deflection in the trace. Interestingly, as shown by the representative calcium uptake traces (Fig. 3a) and by the quantification of maximal calcium capacity (Fig. 3b), TDP-43^A315T^ took up significantly more calcium than N-Tg littermate brain mitochondria. Furthermore, the rate of mitochondrial depolarization, which reflects the proportion of mitochondria that undergo permeability transition upon increasing calcium loads, was slower in TDP-43A^315T^ mitochondria (Fig. 3c).

Western blot analyses of brain mitochondria excluded that the levels of key proteins involved in mitochondrial calcium handling, namely the mitochondrial calcium uniporter (MCU), cyclophilin D (CypD), the MCU regulator MICU1, and the voltage dependent anion channel VDAC1, were unchanged in TDP-43A^315T^ as compared to N-Tg mitochondria (*n* = 8 and 9 for N-Tg and TDP-43A^315T^ respectively, Fig. 3d, e).

Taken together, these results indicated that brain mitochondria of TDP-43 mutant mice had well preserved calcium handling mechanisms, and also suggested that these mitochondria were in fact more resistant to calcium-induced MPTP than N-Tg controls.

### Energy metabolism is unaffected in TDP-43 mutant human skin fibroblasts

In order to test cellular energy metabolism in patient-derived cells expressing physiological levels of mutant TDP-43, we studied primary skin fibroblasts from 3 individuals harboring the N352S point mutation associated with ALS-FTLD [26] and 8 healthy controls, four of whom were kindred of the patients.

**Fig. 4:**
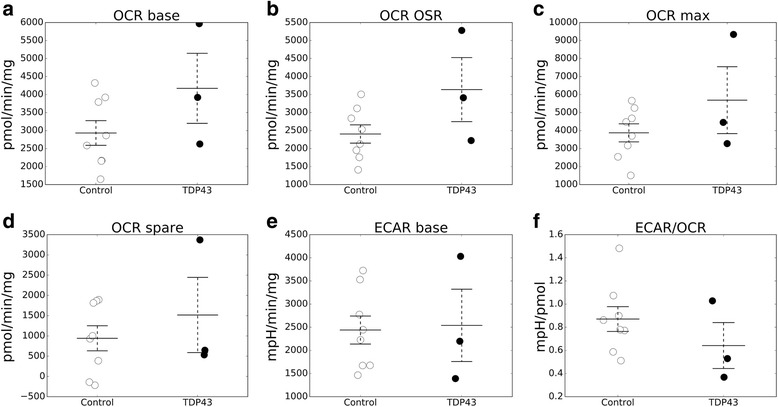
Oxygen consumption and extracellular acidification rates are unchanged in TDP-43 mutant human fibroblasts. **a** Baseline oxygen consumption rate (OCR base) expressed as pmol oxygen consumed per minute per mg of cellular proteins in control skin fibroblasts (*n* = 8 cell lines) and in fibroblasts harboring a TDP-43 N352S point mutation associated with ALS-FTLD (*n* = 3 cell lines). **b** Oligomycin sensitive oxygen consumption rate (OCR OSR) indicating the ATP synthetic component of OCR. **c** Maximal uncoupled respiratory capacity after FCCP addition (OCR max). **d** OCR spare (difference between base and maximum capacity) indicating the reserve respiratory capacity. **e** Baseline extracellular acidification rate (ECAR base), expressed as mpH per minute per mg of cellular proteins, indicating glycolytic flux. **f** ECAR/OCR ratio indicating glycolytic vs. oxidative metabolism. All lines were tested in 12 technical over three biological replicates

Fibroblasts were subjected to flux analyses of oxygen consumption (OCR) and extracellular acidification (ECAR) rates using the Seahorse XF96 platform. Average OCR rates at baseline respiration were not significantly different in TDP-43 and control fibroblasts (Fig. 4a). Similarly, the proton leak (i.e., residual respiration after oligomycin addition, Fig. 4b) and maximal uncoupled respiration (i.e., respiration after FCCP addition, Fig. 4c) were unchanged in patient and control cells. Spare respiratory capacity, indicating the reserve of respiration potential unused under basal conditions, was also not significantly different (Fig. 4d). ECAR, which reflects the anaerobic utilization of glucose and lactate production, was unchanged in TDP-43 mutant cells at baseline (Fig. 4e) and after oligomycin or FCCP additions (data not shown). Importantly, the ECAR:OCR ratio was similar in mutant and control cells (Fig. 4f), suggesting that in TDP-43 mutant cells there was no shift toward glycolytic energy metabolism, a condition typically observed when mitochondria are defective [27].

**Fig. 5:**
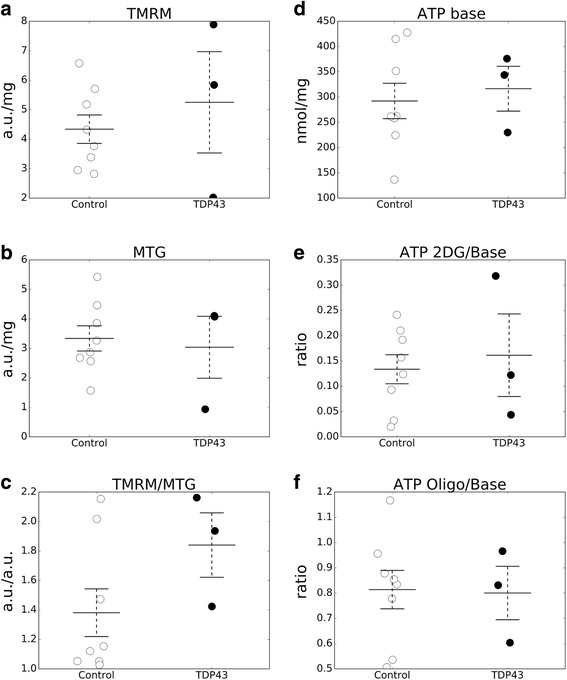
Mitochondrial membrane potential, mitochondrial mass, and ATP content are unchanged in TDP-43 mutant human fibroblasts. **a** Mitochondrial membrane potential estimated fluorimetrically with the potentiometric die TMRM. **b** Mitochondrial mass estimated fluorimetrically with Mito Tracker Green (MTG). **c** Mitochondrial membrane potential normalized by mitochondrial mass (TMRM/MTG). **d** Total cellular ATP content under standard growth conditions. **e** Total cellular ATP content under conditions of glycolytic inhibition. **f** Total cellular ATP content under conditions of mitochondrial ATP synthesis inhibition. *n* = 8 control skin fibroblasts cell lines and *n* = 3 TDP-43 N352S fibroblasts cell lines. All lines were tested in 8 technical over three biological replicates

We then assessed the ability of mitochondria to accumulate the potentiometric fluorescent dye TMRM in TDP-43 mutant fibroblasts and controls. This approach allows for evaluating differences in mitochondrial membrane potential. There were no significant differences in TMRM accumulation between TDP-43 mutant and control cells (Fig. 5a).

Next, to examine the total mitochondrial content we utilized the fluorescent dye MTG, which accumulates in mitochondria in a virtually non-membrane potential dependent manner. We did not detect differences in the mitochondrial content between TDP-43 mutant and control cells (Fig. 5b). Furthermore, to exclude the differences in mitochondrial membrane potential that could be masked by differences in mitochondrial content, we normalized the TMRM values by MTG values. Even when TMRM values were normalized by mitochondrial content (MTG) for each cell analyzed, there were no significant differences between TDP-43 mutant and control cells (Fig. 5c).

Lastly, we analyzed steady state ATP content using a luciferase based assay, which did not show differences between TDP-43 mutant and control fibroblasts under baseline conditions (Fig. 5d). Inhibiting glycolytic ATP synthesis with 2-deoxyglucose resulted in a severe decline of ATP content relative to baseline conditions. The decline had a similar magnitude in TDP-43 mutant and control cells (Fig. 5e). Inhibition of OXPHOS with oligomycin resulted in a more modest decline in ATP content relative to baseline conditions, which did not differ between TDP-43 mutant and control cells (Fig. 5f).

Taken together, these results indicated that energy metabolism in both TDP-43 mutant and control fibroblasts was highly reliant on glycolysis and that there was no significant difference in the ability to run glycolysis or OXPHOS between mutants and controls, thereby excluding that mutant cells had bioenergetic defects.

### Energy metabolism is unaffected in HEK293 cells expressing various TDP-43 mutants

**Fig. 6:**
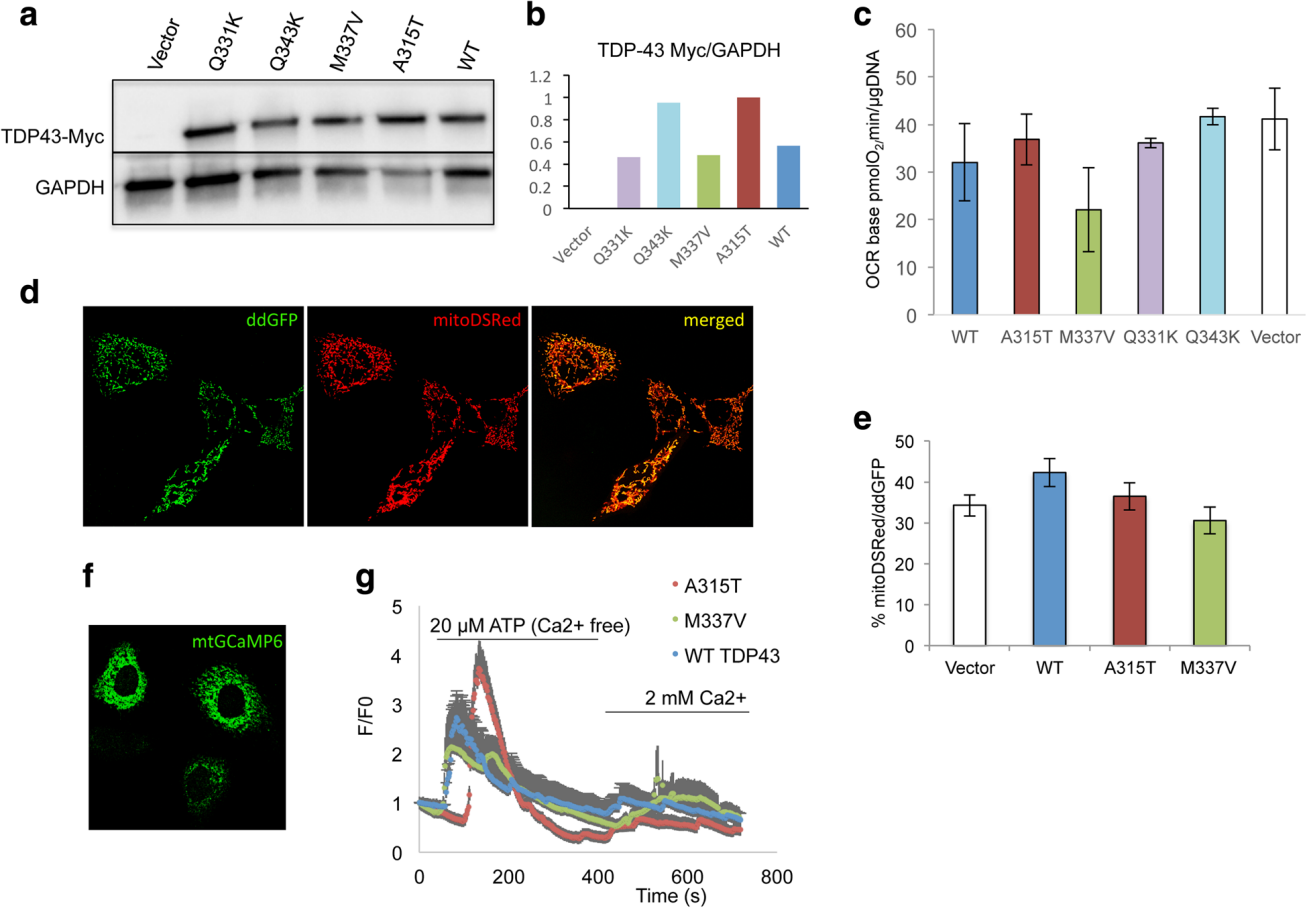
Mitochondrial respiration, ER-mitochondrial contacts, and intracellular calcium handling in HEK293T cells expressing mutant TDP-43. **a** Western blot of cell homogenates from HEK293T cells transiently transfected with constructs expressing recombinant TDP-43-myc probed with myc and GAPDH as a loading control. **b** Quantification of TDP-43 band intensities relative to the intensity of GAPDH (average of two western blots). **c** OCR baseline measurements of HEK293T cells transfected with TDP-43-myc constructs. All lines were tested in 12 technical over three biological replicates. **d** Representative micrographs of HeLa cells co-transfected with the fluorescent reporters ddGFP and mitoDSRed. **e** Mean co-localization of mitoDSRed and ddGFP in HeLa cells expressing WT or mutant TDP-43. *n* = 30–40 cells over three independent transfections. **f** Representative micrograph of HeLa cells transfected with the fluorescent reporter GCamp6 targeted to mitochondria (mtGCamp6). **g** Average mtGCamp6 fluorescence curves indicating mitochondrial calcium dynamics in HeLa cells expressing WT or mutant TDP-43 in response to mobilization of ER calcium stores induced by perfusion with ATP in calcium free buffer, followed by perfusion with 2 mM calcium buffer to induce SOCE. *n* = 12–15 cells over two independent transfections

To extend the analyses of cellular bioenergetics to a larger number of TDP-43 mutations, we expressed recombinant human TDP-43 with the Q331K, Q343K, M337 V, A315T, as well as wild type (WT) TDP-43, in HEK293T cells. Cells were transiently transfected by lipofection with plasmids encoding for various TDP-43 constructs, resulting in similar levels of protein expression for each construct, as determined by western blot analyses of cell homogenates and quantification (Fig. 6a, b). OCR and ECAR were measured by Seahorse flux analyzer in cells transfected with TDP-43 and with empty vector. There were no significant differences in baseline oxygen consumption (Fig. 6c) and acidification rates (data not shown) among cells transfected with the various TDP-43 constructs and empty vector controls. Furthermore, no significant differences were detected between cells expressing TDP-43 and vector controls in OCR and ECAR, when cells were treated with oligomycin, FCCP, or in the ECAR:OCR ratios (data not shown). These results indicated that expression of several forms of mutant or WT TDP-43 did not impair overall energy metabolism in HEK293T cells.

### ER-mitochondria contacts are unaltered, while ER calcium mitochondrial uptake is increased, in cells expressing mutant TDP-43

TDP-43 was shown to alter the physical interactions between mitochondria and ER [13], leading to functional alterations in intracellular calcium handling, which could potentially affect mitochondrial bioenergetics, when calcium is released from internal ER stores. Therefore, we used a dimerization-dependent GFP (ddGFP) to explore the mitochondrial-ER contacts in HeLa cells co-transfected with WT or various TDP-43 mutant constructs. The GFP monomers were targeted to the mitochondrial outer membrane and the ER membrane, respectively. Cells were also transfected with DSred2-Mito to image mitochondria (Fig. 6d). The ddGFP only fluoresces when the monomers are closely apposed, indicating that the ER and mitochondrial membranes are juxtaposed [28]. Quantification of the percentage of co-localization of DSred2-Mito with ddGFP fluorescence (i.e., co-localization of red and green fluorescence), which is an indicator of the proximity of the two organelles, did not differ between cells expressing WT or mutant TDP-43 (M337 V and A315T) and vector only cells (Fig. 6e). This result indicated that, by this assay, mutant TDP-43 expression in HeLa cells did not significantly alter mitochondria-ER contacts.

Next, we determined mitochondrial calcium uptake in TDP-43 expressing cells upon calcium release from the ER, following metabotropic activation of the inositol 3-phosphate receptor through stimulation of purinergic receptors by extracellular ATP. HeLa cells expressing WT or mutant (M337 V and A315T) TDP-43 were transfected with the calcium reporter mtGCaMP6 targeted selectively to the mitochondrial matrix [29] (Fig. 6f). ER calcium release was stimulated by perfusion with 20 μM ATP in calcium-free buffer, followed by perfusion with calcium-containing buffer to activate store-operated calcium entry (SOCE). Interestingly, the mitochondria of A315T TDP-43 expressing cells took up significantly more calcium than both WT and M337 V TDP-43 expressing cells, as indicated by the higher fluorescence peak (Fig. 6g). On the other hand, no difference was observed in mitochondrial calcium uptake when calcium was superfused and entered cells through SOCE mechanisms. Since the physical connections between the ER and mitochondria were unchanged by A315T TDP-43, a likely explanation for this observation is that cells expressing A315T TDP-43 have increased ability to accumulate calcium in mitochondria, which is reminiscent of the increased calcium uptake observed in brain mitochondria of TDP-43^A315T^ mice (Fig. 3).

### TDP-43 association with mitochondria is peripheral, and does not affect the levels of respiratory chain subunits

**Fig. 7:**
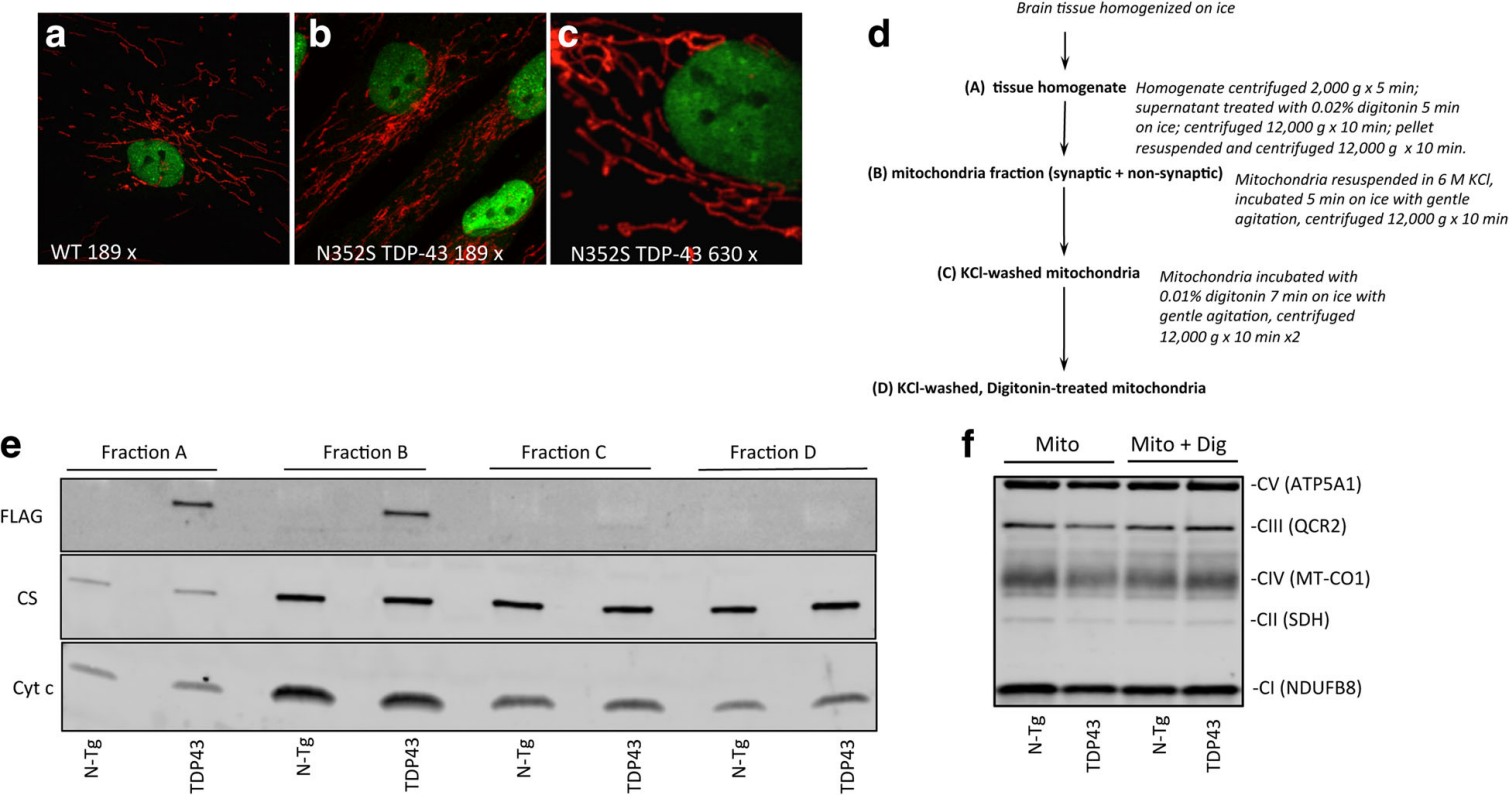
TDP-43 subcellular localization in human fibroblasts and in mitochondrial fractions of mouse brain. **a**-**c** Immunostaining of human WT **a** and TDP-43 N352S **b** fibroblasts cell lines with antibodies against TDP-43 (*in green*) and cytochrome **c** (*in red*) to label mitochondria. Panel **c** shows a higher magnification of TDP-43 N352S fibroblasts. **d** Scheme describing the procedure for mitochondrial isolation, with KCl and digitonin treatments, from mouse brain. **e** Western blot to detect association of recombinant FLAG-tagged TDP-43 with mitochondria obtained from brain of TDP-43^A315T^ mice and N-Tg littermates at 60 days of age. Cytochrome C (Cyt c) and citrate synthase (*CS*) were used as markers of intermembrane space and matrix proteins, respectively. **f** Western blots to detect OXPHOS subunits in percoll-purified brain mitochondrial fractions, prepared without (*Mito*) and with digitonin treatment (*Mito + Dig*) from TDP-43^A315T^ mice and N-Tg littermates

It was proposed that TDP-43, not only is associated with mitochondria, but is also imported inside the organelles due to an internal targeting amino acid signal, and that the mitochondrial import of TDP-43 is increased by the pathogenic mutations both in cells and in the TDP-43^A315T^ mice [14]. To test the hypothesis that TDP-43 is associated with mitochondria we first performed immunostaining of fibroblasts with antibodies directed against TDP-43 and against the mitochondrial protein cytochrome c. In both control and TDP-43 N352S mutant fibroblasts, TDP-43 was detectable exclusively in the nucleus (Fig. 7a-c) and no co-localization of TDP-43 with the mitochondrial marker was observed.

Next, we used a biochemical approach to examine the association of TDP-43^A315T^ with mitochondria in vivo, in mouse brain. Since the transgenic construct contains a FLAG epitope, mitochondrial fraction was analyzed by western blot, using anti-FLAG antibodies. Importantly, we compared the mitochondrial fraction treated with KCl washes, with and without the addition of a low dose of the detergent digitonin to eliminate any potential contamination from non-mitochondrial membranes, such as ER and peroxisomes. Figure 7d summarizes the brain mitochondria preparation steps that were employed in this experiment. An association of TDP-43^A315T^-FLAG with brain mitochondria was clearly detected in the fractions not subjected to KCl washes or digitonin treatment (Fig. 7e). However, immunoreactive bands corresponding to TDP-43^A315T^-FLAG were completely undetectable after KCl washes, irrespective of digitonin treatment.

Neither the matrix protein citrate synthase, nor the inter membrane space protein cytochrome c were significantly affected by KCl washes, with and without digitonin treatment, indicating that the dose of digitonin used did not compromise the integrity of the mitochondrial outer and inner membranes. Taken together, these results suggested that a portion of the protein was peripherally associated with brain mitochondria, presumably through electrostatic interactions.

Because it was proposed that TDP-43 in mitochondria results in downregulation of respiratory chain proteins, specifically complex I [14], we assessed the levels of respiratory chain complex subunits in gradient-purified brain mitochondrial fractions of WT and TDP-43^A315T^, using a specific antibody cocktail. The levels of individual subunits of OXPHOS complexes I-V were unchanged in TDP-43^A315T^ mitochondria, relative to N-Tg controls (Fig. 7f), suggesting that mutant TDP-43 associated with brain mitochondria did not affect respiratory chain subunit levels.

## Discussion

Mitochondrial dysfunction is one of the known pathogenic events associated with ALS. In particular, bioenergetic impairment has been amply described in cellular and mouse models of fALS caused by SOD1 mutations [30] and, more recently, by other genetic forms of the disease, such as mutations in C9Orf72 [31, 32], VCP [33], and CHCHD10 [34]. TDP-43 mutations cause rare cases of fALS, but cytosolic aggregation of wild type TDP-43 in motor neurons is a prominent pathological feature of ALS, including the most prevalent sporadic form of the disease [3]. Mislocalization and aggregation of TDP-43 have been shown to cause abnormalities of mitochondrial morphology and dynamics in cultured neuron systems [35] and in vivo, in peripheral neurons of animal models of TDP-43 ALS [11, 12]. The mechanisms of these abnormalities and their impact on disease pathogenesis remain to be fully elucidated, but mutant TDP-43 was suggested to impair complex I in cultured cells [36]. Moreover, mutant TDP-43 was recently proposed to impair mitochondrial function directly from the matrix compartment, where it causes respiratory chain dysfunction by inhibiting complex I translation [14]. Such mechanism of mitochondrial damage by intra-mitochondrial TDP-43 was proposed in TDP-43^A315T^ transgenic mice and patient-derived cells.

In this study, we sought to validate the findings of bioenergetic dysfunction in TDP-43^A315T^ transgenic mice, patient fibroblasts, and transfected cell expressing TDP-43. Overall, we did not confirm previous findings of mitochondrial bioenergetics defects in any of these models. In particular, mitochondria isolated from the brain of TDP-43^A315T^ transgenic mice did not show impairment of mitochondrial respiration, ATP generation, and calcium handling. Similarly, there was no impairment of bioenergetic functions in skin fibroblasts harboring a pathogenic TDP-43 mutation or in HEK293T cells overexpressing various mutant forms of TDP-43.

We found that a portion of TDP-43 was peripherally associated with the surface of intact mouse brain mitochondria. This association, despite not interfering with mitochondrial bioenergetics, could affect inter-organellar communication. Indeed, TDP-43 was shown to interfere with the structures that link mitochondria and ER, also known as mitochondria associated membranes or MAMs [37]. Our ddGFP assay, did not detect an overall change in the amount of ER-mitochondrial contacts, but this method does not specifically detect MAMs. Therefore, more work, using a variety of experimental approaches, which are beyond the scope of this study on mitochondrial function, will have to be done to further explore the effects of the peripheral association of TDP-43 with mitochondria.

Earlier studies had shown that TDP-43 overexpression alters mitochondrial dynamics in neurons, resulting in decreased motility accompanied by abnormal clustering and fragmentation [10, 12, 38]. Based on our results showing that mitochondrial bioenergetics is essentially unaffected by mutant TDP-43, we conclude that the mitochondrial dynamics abnormalities are not the result of intrinsic mitochondrial bioenergetics defects or intramitochondrial localization of mutant TDP-43. It is logical to speculate that abnormal mitochondrial movement and morphology could be caused by alterations in components of the mitochondrial dynamics machinery, which are localized in extra-mitochondrial compartments or on the outer mitochondrial membrane. This could lead to impaired motility and mitochondrial clustering along the neuronal processes. Dysregulation of mitochondrial transport by mutant TDP-43 could impair the correct positioning of mitochondria relative to major sites of energy utilization, such as synapses. For example, the levels of the outer membrane cargo adaptor Miro1, a protein necessary for the docking of mitochondria on microtubule tracks, were decreased in spinal cord of mice expressing mutant TDP-43 as well as ALS patients [35]. We propose that TDP-43 causes alterations of proteins involved in mitochondrial transport, such as Miro1, or components of the fusion and fission apparatus, without TDP-43 internalization in mitochondria or bioenergetic defects.

Interestingly, brain mitochondrial calcium uptake capacity was increased in TDP-43^A315T^ transgenic mice relative to controls. This result suggests that TDP-43 A315T mitochondria were less prone to undergo calcium induced permeability transition than controls, a further indication that they did not suffer from bioenergetic impairment.

Like the mouse model, cultured cells expressing mutant TDP-43 did not show bioenergetic defects, both when endogenous levels of the protein were present, in patient-derived fibroblasts, and when the mutant proteins were overexpressed, in transfected cells. The results in fibroblasts were in line with a recent report, which compared C9Orf72 mutant and TDP-43 mutant fibroblasts, and detected respiratory chain defects in the former, but not the latter [31]. Furthermore, similar to the transgenic mouse brain, cells expressing A315T TDP-43 had increase mitochondrial calcium uptake upon ER calcium release.

The reasons for the discrepancy between the present study and a recent report in which mitochondrial respiratory chain defects were associated with mitochondrial import of mutant TDP-43 [14] is not immediately clear, especially in regards to the TDP-43^A315T^ mouse model, since we utilized mice from the same transgenic line and at similar ages as in the aforementioned report. It is possible that differences resulted from the protocols of mitochondrial purification, which were partly dissimilar in the two studies. It could be hypothesized that mitochondria from mutant TDP-43 brains are more fragile and could be damaged by the isolation procedure, which was not the case in our study, because soluble proteins of the intermembrane space were retained in the mitochondrial preparation. On the other hand, membrane fragility could not explain a decline in the activity of individual respiratory chain complexes, such as complex I, whose activities are measured on fractionated mitochondrial membranes and do not depend on mitochondrial integrity. Specifically, we did not find evidence of a decrease in either complex I-driven respiration and ATP synthesis or complex I oxidoreductase activity in brain mitochondria from TDP-43^A315T^ transgenic mice, suggesting that OXPHOS is preserved in these mitochondria. Furthermore, the levels of respiratory chain subunits that we analyzed were unchanged in TDP-43^A315T^ transgenic mouse brain mitochondria, suggesting that their synthesis was not impaired.

Unlike previous reports [14, 36], we did not detect respiratory defects in mutant TDP-43 human fibroblasts. A possible explanation for this discrepancy in findings could be ascribed to the different TDP-43 mutations investigated in previous studies and the present one, since we analyzed lines from three patients from one family with the N352S mutation and their healthy relatives. Nevertheless, we also tested the effects of recombinant mutant TDP-43 expression in HEK293T cells, an approach similar to the one utilized previously [14], but did not detect respiratory defects in these mutant cells either.

## Conclusions

Multiple lines of evidence support a role for mutant TDP-43 in causing alterations of mitochondrial motility, morphology, and distribution in neurons, which could result in failure to provide energy to critical sites of utilization. However, our present studies did not confirm reports of mutant TDP-43 causing impaired mitochondrial bioenergetics in vivo and in cultured cells, suggesting that, at least in the systems we investigated, mitochondrial OXPHOS dysfunction does not directly participate in disease pathogenesis. On the other hand, we report the intriguing finding of increased mitochondrial calcium uptake in mouse brain mitochondria and patient derived fibroblasts harboring A315T TDP-43. The pathological significance and especially the mechanisms underlying this observation remain to be determined, but they do not appear to involve changes in mitochondrial bioenergetics.

## Abbreviations

AA: Antimycin A
ALS: Amyotrophic lateral sclerosis
BSA: Bovine serum albumin
CypD: Cyclophilin D
ddGFP: Dimerization dependent GFP
ECAR: Extracellular acidification rate
ER: Endoplasmic reticulum
fALS: Familial ALS
FCCP: Carbonyl cyanide p-trifluoromethoxyphenylhydrazone
FTLD: Frontotemporal lobar degeneration
HM: Homogenization buffer
MCU: Mitochondrial calcium uniporter
MTG: Mito Tracker Green
OCR: Oxygen consumption rate
OXPHOS: Oxidative phosphorylation
Rot: Rotenone
SOCE: Store-operated calcium entry
SOD1: Superoxide dismutase 1
TDP-43: Transactivation response element DNA-binding protein 43
TMRM: Tetramethylrhodamine methyl ester
WT: Wild type
